# Clinical significance of DNA methylation mRNA levels of *TET* family members in colorectal cancer

**DOI:** 10.1007/s00432-014-1901-2

**Published:** 2015-01-04

**Authors:** Agnieszka Anna Rawłuszko-Wieczorek, Agnieszka Siera, Karolina Horbacka, Nikodem Horst, Piotr Krokowicz, Paweł Piotr Jagodziński

**Affiliations:** 1 Department of Biochemistry and Molecular Biology, Poznań University of Medical Sciences, Poznan, Poland; 2 Department of General and Colorectal Surgery, Poznań University of Medical Sciences, Poznan, Poland

**Keywords:** DNA methylation, TET enzymes, Prognostic factor, Colorectal cancer

## Abstract

**Purpose:**

Ten eleven translocation (TET) enzyme activity is essential for active DNA demethylation in biological processes, and their altered expression has been observed in various malignancies. Therefore, we investigated DNA methylation and mRNA levels of all *TETs* in colorectal cancer (CRC) patients.

**Methods:**

*TET* mRNA levels were evaluated using quantitative RT-PCR in primary cancerous and histopathologically unchanged colorectal tissues from patients who underwent radical surgical colon resection (*n* = 113). DNA methylation levels of the *TET* CpG island were assessed using bisulfite DNA sequencing and high-resolution melting analysis.

**Results:**

We found reduced transcript levels of TET1, TET2 and TET3 in cancerous tissue compared with their histopathologically unchanged counterparts (*p* = 0.000011; *p* = 0.000001; *p* = 0.00031, respectively). Importantly, multivariate Cox regression analysis revealed favorable overall survival (OS) and disease-free survival (DFS) outcomes for patients with high TET2 mRNA levels in histopathologically unchanged tissue (HR^OS^ = 0.091, 95 % CI 0.011–0.77, *p* = 0.028; HR^DFS^ = 0.21, 95 % CI 0.04–1.06, *p* = 0.059). Moreover, we found no DNA methylation in the *TET2* and *TET3* promoter regions in cancerous and histopathologically unchanged tissue. In contrast, we reported *TET1* DNA hypermethylation in a small fraction of patients (*n* = 12/113).

**Conclusion:**

To best of our knowledge, our study is the first to investigate TET mRNA levels in a cohort of CRC patients and correlate them with patients’ prognosis. Present study provides the evidence that TET2 mRNA expression may be an independent prognostic factor for disease recurrence and outcome. Additionally, our findings initially indicate the importance of DNA methylation in regulating *TET1* expression.

**Electronic supplementary material:**

The online version of this article (doi:10.1007/s00432-014-1901-2) contains supplementary material, which is available to authorized users.

## Introduction

Colorectal cancer (CRC) growth is influenced by genetic and epigenetic abnormalities. The most extensively characterized epigenetic modification is methylation at the 5-carbon of cytosines, mostly in the context of CG dinucleotides. Aberrant patterns of DNA methylation are observed from the early stages of CRC carcinogenesis (Dawson and Kouzarides 2012). DNA hypermethylation within promoter regions leads to the inactivation of CRC tumor suppressor genes, while hypomethylation is associated with genomic instability (Migliore et al. 2011). DNA methyltransferases (DNMTs), which establish and maintain DNA methylation patterns, are responsible for silencing tumor suppressor genes; thus, they are thought to be oncogenes (Linhart et al. 2007). However, the role of the enzymes involved in DNA demethylation in CRC remains elusive.

In 2009, two research groups identified independently ten eleven translocation 1 (TET1) activity to convert 5-methylcytosine (5-mC) to 5-hydroxymethylcytosine (5-hmC) (Ito et al. 2010; Tahiliani et al. 2009). Soon after, the TET2 and TET3 family members were characterized (Ito et al. 2010). Moreover, studies revealed that the TET proteins further convert 5-hmC to 5-formylocytosine (5-fC) and 5-carboxycytosine (5-caC) (He et al. 2011; Ito et al. 2011). As a result, it has been suggested that the TET proteins participate in DNA demethylation processes through at least four mechanisms: replication-dependent passive dilution of 5-hmC; removal of oxidative cytosines by thymine-DNA glycosylase (TDG) and base excision repair system (BER); activation-induced deaminase/apolipoprotein B mRNA-editing enzyme complex (AID/APOBEC), which may deaminate 5-hmC to 5-hydroxyuracyl (5-hmU) that is then removed by TDG; and decarboxylation of 5-caC to an unmodified cytosine without BER activity (Guo et al. 2011; Hashimoto et al. 2012; Schiesser et al. 2012; Stivers and Jiang 2003).

Recent studies have indicated lower level of 5-hmC and *TET* expression in many malignancies compared with normal tissues (Frycz et al. 2014; Lian et al. 2012; Liu et al. 2013; Muller et al. 2012). However, only one article ascertained the expression of *TET* gene family members in CRC in a small subset of patients (*n* = 22) (Kudo et al. 2012). Moreover, although the initial data indicates posttranscriptional and posttranslational control of *TET* expression, relatively little is known about their transcriptional control (Delatte et al. 2014). Interestingly, all *TET* promoter regions contain CpG islands that can potentially undergo silencing by DNA methylation. Therefore, in this study, we investigated alterations in DNA methylation and mRNA levels of *TET1*, *TET2* and *TET3* in primary cancerous and histopathologically unchanged colorectal tissues from a cohort of 113 patients. The results obtained were correlated with clinicopathological factors and prognostic significance.

## Materials and methods

### Patient material

Primary colonic adenocarcinoma tissues were collected between June 2009 and March 2013 from a cohort of 113 patients who underwent radical surgical resection of the colon at the Department of General and Colorectal Surgery, Poznań University of Medical Sciences, Poland (Table 1). Histopathologically unchanged colonic mucosa located at least 10–20 cm away from the cancerous lesions were obtained from the same patients. One set of samples was immediately snap-frozen in liquid nitrogen and stored at −80 °C until DNA and RNA isolation. The other set of samples was directed for histopathological examination performed by an experienced pathologist. None of the patients received preoperative chemo- or radiotherapy. Informed consent was obtained from all participating individuals. The procedures of the study were approved by the Local Ethical Committee of Poznań University of Medical Sciences. Written consent was obtained from all participants.

**Table 1:** Clinicopathological characteristics of patients with colorectal cancer

Features	No. of patients
Total no. of patients	113
Gender (female/male)	47/66
Mean (±SD) age at radical surgical resection of colon (years)	66.84 ± 11.65
CRC localization	
Proximal colon (cecum to transverse)	39
Distal colon (splenic flexure to sigmoid)	21
Rectum	53
Histological grade	
G1	7
G2	70
G3	36
TNM classification	
I	17
IIA	40
IIC	6
IIIA	3
IIIB	34
IIIC	13

### Measurement of overall and disease-free survival

Follow-up data were available for 88 patients, who were observed from 2009/08/01 until death or 2014/25/05, whichever came first. Disease-free survival (DFS) is defined as the time elapsed from surgery to the first occurrence of any of the following events: recurrence or distant metastasis of CRC or the development of a second non-colorectal malignancy. The diagnosis of recurrence and metastasis was evaluated with a variety of methods, including computed tomography, ultrasonography, position emission tomography, cytologic analysis or biopsy. Overall survival (OS) is defined as the time elapsed from surgery to the death of the CRC patient. Death of patients was verified by public records and family reports. The DFS and OS statuses were ascertained by a physician blinded to the TET mRNA levels.

### DNA isolation and bisulfite modification

Genomic DNA from the tissues of patients with CRC was isolated using the DNA Mammalian Genomic Purification Kit purchased from Sigma-Aldrich Co. (St. Louis, MO). Then, 500 ng of genomic DNA was subjected to bisulfite conversion of cytosine to uracil, according to the EZ DNA Methylation Kit™ procedure from Zymo Research Corporation **(**Orange, CA). The positions of the CpG islands and transcription factor-binding sites located in the *TET1, TET2* and *TET3* promoters were determined by online programs (CpGPlot/CpGReport/Isochore; Searcher; Site).

### DNA methylation evaluation by bisulfite sequencing

The DNA fragments containing CpG dinucleotides located in the promoter region of the *TET1*, *TET2* and *TET3* genes were amplified by the primer pairs complementary to the bisulfite-DNA modified sequences (Supplementary Table 1). PCR amplification was performed by FastStart Taq DNA Polymerase from Roche Diagnostic GmbH (Mannheim, Germany). The PCR products were purified using Agarose Gel DNA Extraction Kit, Roche Diagnostic GmbH (Mannheim, Germany) with subsequent cloning into pGEM-T Easy Vector System I, Promega (Madison, WI) and transformation into TOPO10 *E. coli* strain cells. The plasmid DNA isolated from five positive bacterial clones was used for commercial sequencing of the cloned fragment of DNA. The results of bisulfite sequencing were assessed and presented using BiQ analyzer software and bisulfite sequencing data presentation and compilation (BDPC) web server, respectively (Bock et al. 2005; Rohde et al. 2008).

### DNA methylation assessment by high-resolution melting (HRM) analysis

The methylation levels of DNA fragments located within the CpG islands of the *TET1*, *TET2* and *TET3* genes were determined by real-time PCR amplification of bisulfite-treated DNA, followed by HRM profile analysis by Light Cycler^®^480 or LightCycler^®^96 Real-Time PCR System, Roche Diagnostics GmbH (Mannheim, Germany). For PCR amplification, 1 μl of either the bisulfite-treated DNA from patients or standards, together with primers (Supplementary Table 1), was added to 19 μl of 5 X Hot FIREPol EvaGreen HRM Mix, Solis BioDyne Co. (Tartu, Estonia). Standardized solutions with a given DNA methylation percentage were prepared by mixing methylated and non-methylated bisulfite-treated DNA from the Human Methylated/Non-methylated DNA Set, Zymo Research Corp. (Orange, CA) in different ratios. To determine the percentage of methylation, the HRM profiles of patients’ DNA PCR products were compared with HRM profiles of standard DNA PCR products (Rawluszko et al. 2011; Wojdacz and Dobrovic 2007). HRM methylation analysis was performed using the Light Cycler^®^480 or LightCycler^®^96 Gene Scanning software, Roche Diagnostics GmbH (Mannheim, Germany). Each PCR amplification and HRM profile analysis was performed in triplicate. The methylation status for each patient was presented as a percentage of methylation in amplified fragments located in the CpG islands of *TET1*, *TET2* and *TET3*.

### RNA isolation, reverse transcription and real-time quantitative polymerase chain reaction (RQ-PCR) analysis

Total RNA from the tissues of patients with CRC was isolated according to the method of Chomczyński and Sacchi (1987). The RNA samples were quantified and reverse-transcribed into cDNA. RQ-PCR was carried out in the Light Cycler^®^480 Real-Time PCR System, Roche Diagnostics GmbH (Mannheim, Germany) using SYBR^®^ Green as the detection dye. The target cDNA was quantified by the relative quantification method using a calibrator for the primary tissues. The calibrator was prepared as a cDNA mix from all of the patients’ samples, and successive dilutions were used to create a standard curve as described in Relative Quantification Manual, Roche Diagnostics GmbH (Mannheim, Germany). For amplification, 1 μl of (total 20 μl) cDNA solution was added to 9 μl of IQ™ SYBR^®^ Green Supermix, Bio-Rad Laboratories Inc. (Hercules, CA) with primers (Supplementary Table 1). To prevent the amplification of sequences from genomic DNA contamination, primers and/or amplicons were designed at exon/exon boundaries and covered all gene splice variants. The quantity of TET1, TET2 and TET3 transcripts in each sample was standardized by the geometric mean of two internal controls: *porphobilinogen deaminase* (*PBGD*) and *human mitochondrial ribosomal protein L19* (*hMRPL19*) (Supplementary Table 1). The selection of internal control genes was performed as previously described (Rawluszko-Wieczorek et al. 2014). The TET1, TET2 and TET3 transcript levels in the patients’ tissues were expressed as a multiplicity of the cDNA concentrations in the calibrator. A few samples, in which the quantity of isolated RNA was insufficient to prepare high-quality RQ-PCR products, were excluded from further analysis. Hence, the number of patients in the subgroups of survival analysis may vary by ±4 samples.

### Statistical analysis

The normality of the observed patient data distribution was assessed using the Shapiro–Wilk test, and the median values were compared using the *U* Mann–Whitney test. Survival curves were plotted using the Kaplan–Meier method, and survival differences were determined using the log rank test. The multivariable Cox proportional hazard model was used to estimate the adjusted hazard ratio (HR). Statistical analysis was performed with the STATISTICA 10.0 software, and *p* < 0.05 was considered statistically significant.

## Results

### mRNA levels of *TET* family members are significantly decreased in cancerous tissues compared with histopathologically unchanged tissues from patients with CRC

**Fig. 1:**
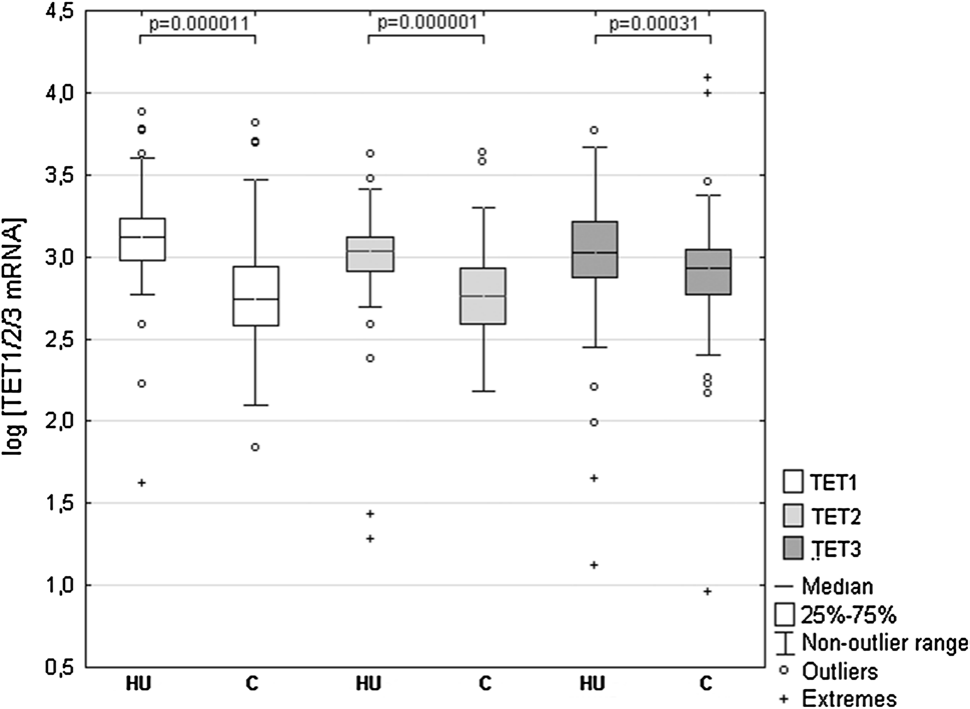
TET1, TET2 and TET3 transcript levels in primary cancerous and histopathologically unchanged tissues from patients with CRC. The cancerous (*C*) and histopathologically unchanged tissues (*HU*) from 113 patients with CRC were used for RNA isolation. Total RNA was reverse-transcribed, and cDNAs were investigated by RQ-PCR relative quantification analysis. The TET1, TET2 and TET3 mRNA levels were corrected by the geometric mean of PBGD and hMRPL19 cDNA levels. The amounts of TET1, TET2 and TET3 mRNA were expressed as the decimal logarithm of multiples of these cDNA copies in the calibrator

We used RQ-PCR to compare TET1, TET2 and TET3 transcript levels in cancerous and histopathologically unchanged tissues in 113 patients with CRC. We found significantly lower levels of TET1, TET2 and TET3 mRNA levels (*p* = 0.000011; *p* = 0.000001; *p* = 0.00031, respectively) in cancerous tissues than in histopathologically unchanged tissues (Fig. 1). The lower levels of TET1 and TET2 mRNA levels in cancerous tissues were observed across different age groups, genders, CRC localization, histological grades and TNM classification (Table 2A, B). Differences in the IIC and IIIA TNM classes did not reach statistically significant results for all the TETs, most likely due to the small number of patients in these subgroups. However, age and localization-related expression was observed for the TET3 transcript. Significantly lower TET3 mRNA levels in the cancerous tissues was observed in the groups of patients above 60 years of age, and in tumors localized in the proximal colon (Table 2C).

**Table 2:** TET1 (A), TET2 (B) and TET3 (C) transcript levels in primary cancerous and histopathologically unchanged tissue samples from patient with CRC

	Primary cancerous tissue	Histopathologically unchanged tissue	*p* ^a^
	Mean (range)		
*A. TET1*			
	2.79 (1.84–3.81)	3.13 (1.62–3.88)	0.000001
Age (years)			
<60	2.78 (2.36–3.70)	3.09 (1.62–3.54)	0.0036
>60	2.78 (1.84–3.81)	3.14 (2.23–3.89)	0.000001
Gender			
Female	2.79 (1.84–3.35)	3.16 (2.23–3.89)	0.000001
Male	2.77 (2.10–3.81)	3.11 (1.62–3.78)	0.000001
Localization			
Proximal colon	2.69 (1.84–3.81)	3.16 (1.62–3.88)	0.000001
Distal colon	2.77 (2.35–3.29)	3.16 (2.85–3.78)	0.000016
Rectum	2.85 (2.20–3.70)	3.09 (2.23–3.62)	0.000043
Histological grade			
G1	2.83 (2.57–3.03)	3.18 (2.91–3.43)	0.022
G2	2.76 (1.84–3.70)	3.13 (2.23–3.78)	0.000001
G3	2.81 (2.10–3.81)	3.11 (1.62–3.88)	0.00084
TNM classification			
I	2.77 (2.54–3.04)	3.15 (2.77–3.78)	0.00023
IIA	2.68 (1.84–3.70)	3.13 (1.62–3.76)	0.000004
IIC	2.70 (2.10–3.17)	3.25 (3.07–3.60)	0.21
IIIA	2.76 (2.58–2.94)	3.18 (2.84–3.27)	0.92
IIIB	2.84 (2.14–3.81)	3.07 (2.23–3.88)	0.0039
IIIC	2.84 (2.46–3.28)	3.21 (2.95–3.54)	0.00028
*B. TET2*			
	2.76 (2.18–3.63)	3.03 (1.28–3.63)	0.000001
Age (years)			
<60	2.76 (2.30–3.18)	2.98 (1.28–3.26)	0.0057
>60	2.76 (2.18–3.63)	3.03 (1.43–3.63)	0.000001
Gender			
Female	2.75 (2.34–3.23)	3.01 (2.71–3.40)	0.000001
Male	2.76 (2.18–3.63)	3.03 (1.28–3.63)	0.000001
Localization			
Proximal colon	2.71 (2.28–3.58)	3.04 (1.28–3.42)	0.000001
Distal colon	2.68 (2.38–3.07)	3.05 (2.78–3.48)	0.000014
Rectum	2.79 (2.18–3.63)	2.97 (1.43–3.63)	0.00002
Histological grade			
G1	2.69 (2.22–3.06)	3.02 (2.72–3.26)	0.21
G2	2.74 (2.18–3.23)	3.01 (1.43–3.63)	0.000001
G3	2.79 (2.30–3.63)	3.04 (1.28–3.39)	0.00025
TNM classification			
I	2.73 (2.46–3.00)	2.98 (2.72–3.63)	0.0035
IIA	2.64 (2.30–3.23)	3.04 (1.28–3.42)	0.000002
IIC	3.00 (2.66–3.07)	3.07 (2.92–3.11)	0.38
IIIA	2.79 (2.70–2.87)	2.91 (2.81–3.00)	0.69
IIIB	2.79 (2.18–3.63)	2.97 (1.43–3.26)	0.00062
IIIC	2.76 (2.44–3.03)	3.03 (2.76–3.13)	0.0017
*C. TET3*			
	2.93 (0.96–4.08)	3.03 (1.12–3.77)	0.00031
Age (years)			
<60	2.96 (2.17–3.99)	3.02 (1.65–3.66)	0.13
>60	2.91 (0.95–4.08)	3.03 (1.12–3.77)	0.0013
Gender			
Female	2.92 (2.22–3.99)	3.04 (2.49–3.77)	0.025
Male	2.92 (0.96–4.08)	3.01 (1.12–3.59)	0.0065
Localization			
Proximal colon	2.90 (2.17–4.08)	3.07 (1.65–3.77)	0.00034
Distal colon	2.88 (2.22–3.28)	2.95 (2.56–3.66)	0.26
Rectum	2.94 (0.96–3.99)	2.97 (1.12–3.46)	0.36
Histological grade			
G1	3.06 (2.91–3.19)	3.08 (2.78–3.46)	0.99
G2	2.90 (0.96–3.45)	3.02 (1.99–3.77)	0.00053
G3	2.94 (2.17–4.08)	3.03 (1.12–3.59)	0.078
TNM classification			
I	2.94 (2.22–3.28)	3.12 (1.99–3.59)	0.064
IIA	2.94 (2.17–3.99)	3.02 (1.65–3.77)	0.069
IIC	2.84 (2.42–3.23)	3.16 (2.56–3.26)	0.66
IIIA	2.89 (2.84–2.94)	2.97 (2.88–3.06)	0.70
IIIB	2.89 (0.96–4.08)	2.98 (1.12–3.49)	0.029
IIIC	3.00 (2.84–3.37)	3.03 (2.82–3.66)	0.95

The TET1, TET2 and TET3 transcript levels were standardized by the geometric mean of PBGD and hMRPL19 cDNA and expressed as decimal logarithm of multiples of these cDNA copies in calibrator

^a^ *U* Mann–Whitney test

### DNA methylation level of promoter region of *TET* genes

**Fig. 2:**
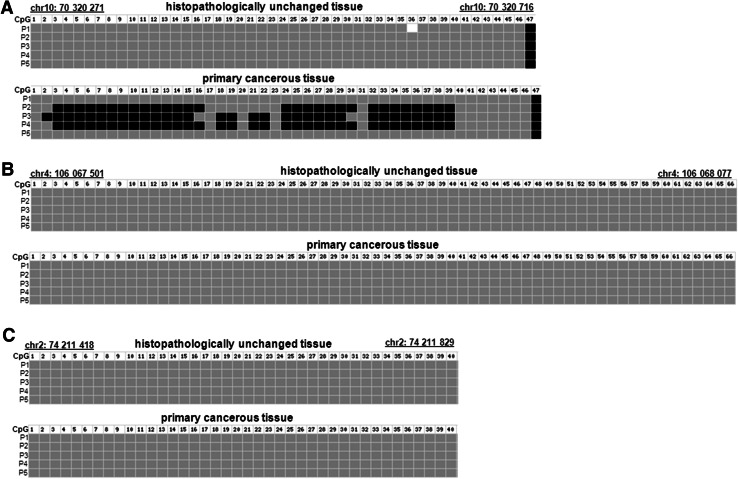
DNA methylation assessment of *TET1*, *TET2* and *TET3* gene regulatory region by bisulfite sequencing in tissue samples from patients with CRC. Primary cancerous and histopathologically unchanged tissues from the same patients with CRC (P1–P5) were used for genomic DNA isolation followed by bisulfite conversion of cytosine to uracil. The TET1, TET2 and TET3 regions containing 47, 64 and 40 CpG dinucleotides, respectively, were then amplified by a pair of primers complementary to the bisulfite-DNA modified sequence. The PCR products were purified with subsequent cloning into a plasmid vector. Plasmid DNA isolated from five positive bacterial clones was used for commercial sequencing. The results of bisulfite sequencing were assessed and presented using BiQ analyzer software and BDPC web server. *Black*, *gray* and *white* *boxes* represent methylated, unmethylated or undetermined CpG dinucleotide, respectively

**Fig. 3:**
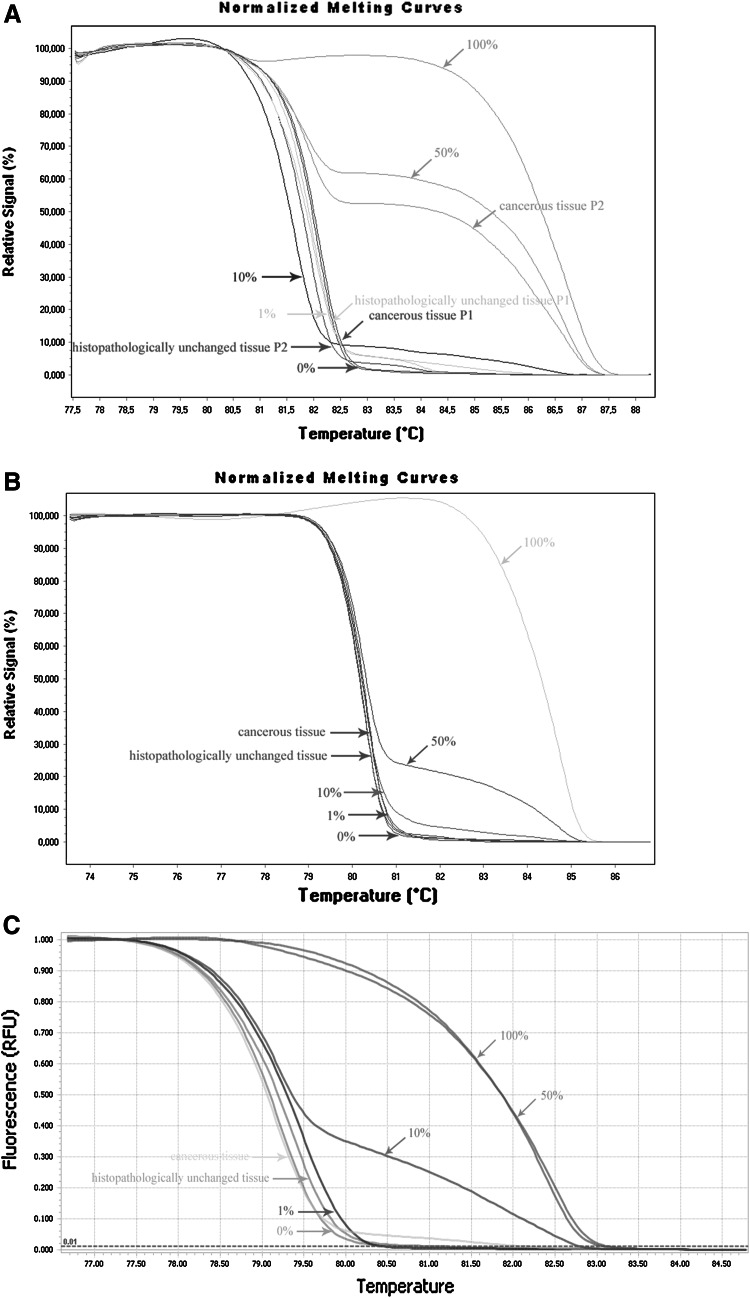
DNA methylation assessment of *TET1*, *TET2* and *TET3* gene regulatory region by HRM analysis in tissue samples from patients with CRC. Primary cancerous and histopathologically unchanged tissues from the same patients with CRC were used for genomic DNA isolation followed by bisulfite conversion of cytosine to uracil. **a**–**c** Represent HRM profiles of standard and example of patient DNA PCR product for TET1, TET2 and TET3, respectively. Methylation percentage of DNA fragments within the CpG island was determined by real-time PCR amplification of bisulfite-treated standard and patient DNA, followed by comparison of their HRM profiles. DNA standards were prepared by mixing different ratios of methylated and non-methylated bisulfite-treated standard DNA. HRM methylation analysis was performed using Light Cycler^®^480 or LightCycler^®^96 Gene Scanning software, Roche Diagnostics GmbH (Mannheim, Germany). Each PCR amplification and HRM profile analysis was performed in triplicate

**Fig. 4:**
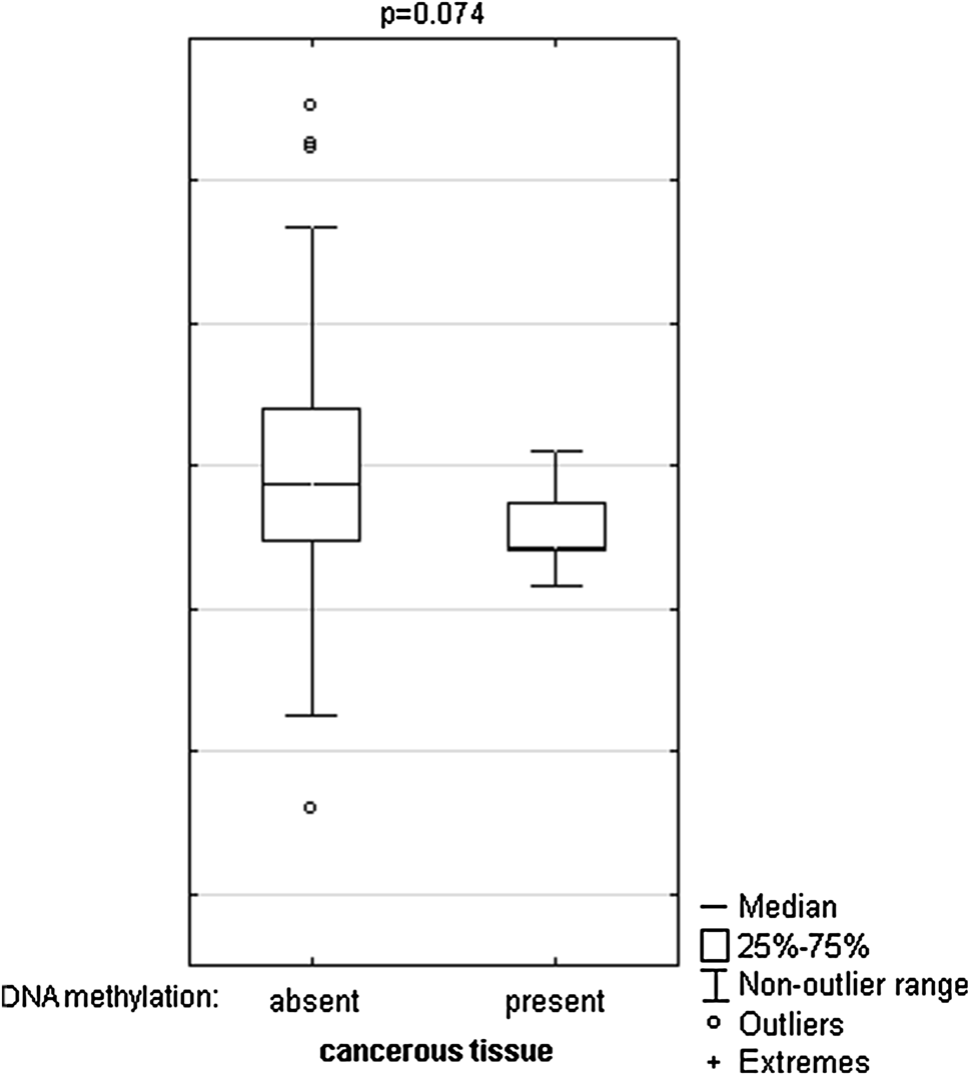
DNA methylation effect on TET1 mRNA levels in cancerous tissue. The primary cancerous tissues from 113 patients with CRC were used for RNA isolation. Total RNA was reverse-transcribed, and cDNAs were investigated by RQ-PCR relative quantification analysis. The TET1 mRNA levels were corrected by the geometric mean of PBGD and hMRPL19 cDNA levels. The amount of TET1 mRNA was expressed as the decimal logarithm of multiples of cDNA copies in the calibrator

To compare DNA methylation levels in the *TET1*, *TET2* and *TET3* promoter regions between DNA samples from the cancerous and histopathologically unchanged tissues, we performed bisulfite DNA sequencing followed by HRM analysis. Bisulfite sequencing was used for evaluation of DNA methylation in large regions of CpG islands in randomly selected patients. We detected a similar pattern of DNA methylation within all individual clones of each patient. We undetected DNA methylation of *TET2* (chr4: 106,067,501–106,068,077) and *TET3* (chr2: 74,211,418–74,211,829) promoter regions using bisulfite sequencing in selected patients (Fig. 2b, c). In keeping with bisulfite sequencing data, we have not observed DNA methylation within *TET2* (chr4: 106 067,537–106,067 735) and *TET3* (ch42: 74,211,418–74,211 584) promoter regions in 113 patients with CRC (Fig. 3b, c). Although HRM analysis revealed no DNA methylation in *TET1* promoter region (chr10: 70 320 271–70,320 457) of 101 samples, significant DNA hypermethylation in cancerous tissues, compared to histopathologically unchanged tissues, was observed in 12 patients (Fig. 3a). The DNA hypermethylation of *TET1*-selected region was also observed using bisulfite sequencing (Fig. 2a). Patients numbered P2–P4 are among group with detected DNA methylation in cancerous tissue using HRM analysis (Fig. 2a). Stratification of the patients with increased DNA methylation in the *TET1* promoter by gender, age, localization, histological grade and TNM did not reveal any significant tendency (Table 3). Nonetheless, patients with DNA hypermethylation in the *TET1* regulatory region of cancerous tissues have lower TET1 transcript levels (*p* = 0.074), compared to cancerous tissues with no DNA methylation changes in the same region (Fig. 4).

**Table 3:** Clinicopathological characteristics of CRC patients with *TET1* DNA hypermethylation in cancerous tissue

Characteristics	No. of cases with *TET1* DNA hypermethylation in cancerous tissue
Age (<60/>60)	2/10
Gender (female/male)	4/8
Localization (proximal/distal/rectum)	5/4/3
Histological grade (G1/G2/G3)	2/7/3
TNM (I/IIA/IIC/IIIB)	3/5/1/3

### TET2 mRNA levels have prognostic potential in CRC patients’ overall and disease-free survival

**Fig. 5:**
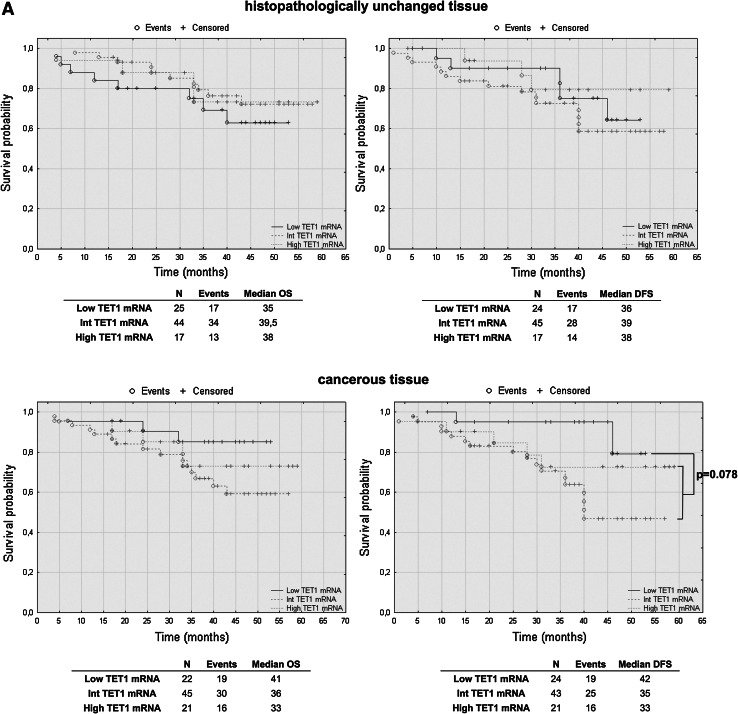

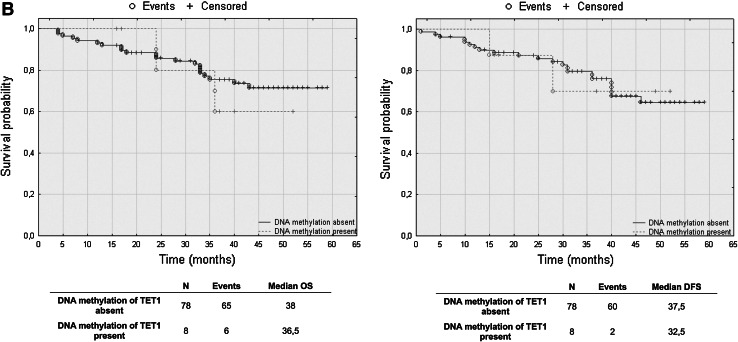

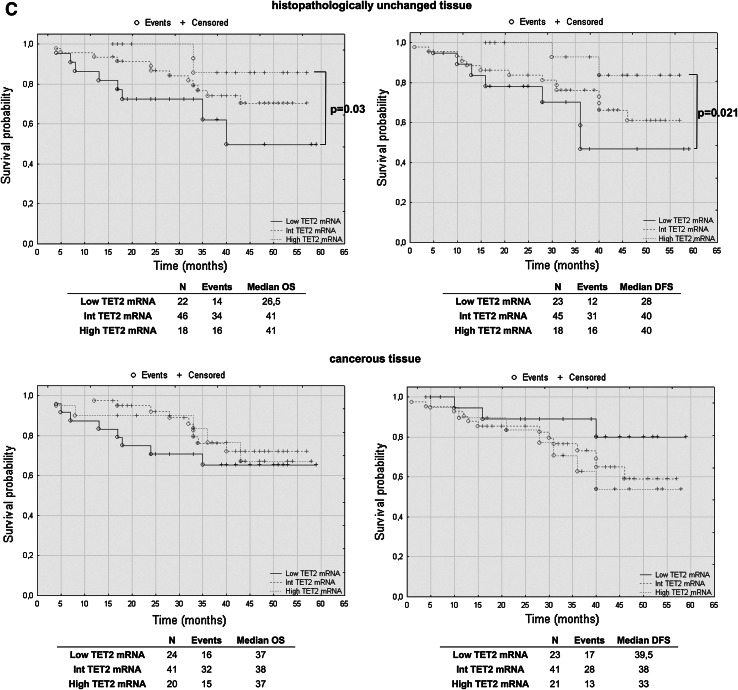

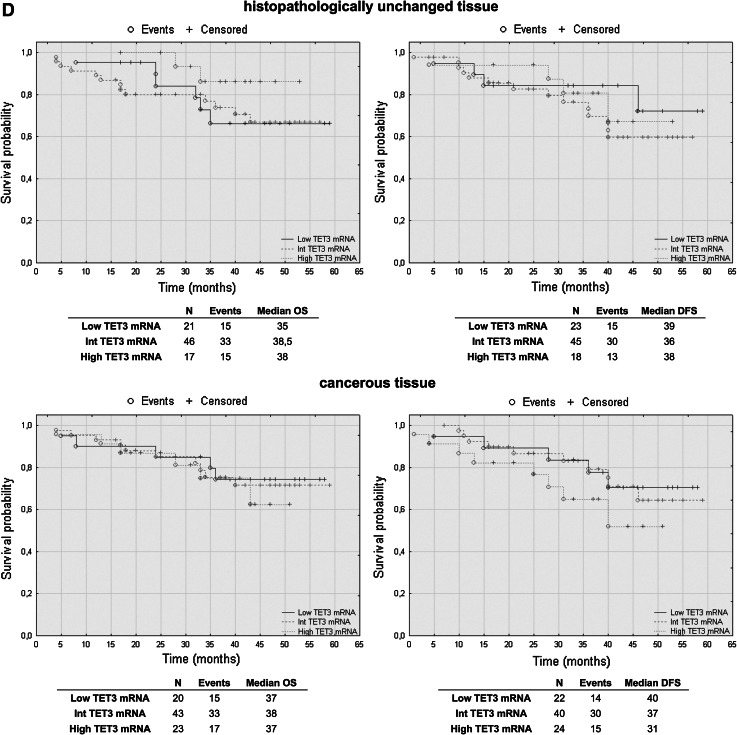
The Kaplan–Meier survival analysis among patients with colorectal cancer according to the TET1 mRNA level (**a**), *TET1* DNA methylation (**b**), TET2 (**c**) and TET3 (**d**) mRNA level. Patients were subdivided into three groups: low, intermediate, and high for each TET1, TET2 and TET3 transcript levels in histopathologically unchanged and cancerous tissue or DNA methylation absent/present in *TET1* promoter in cancerous tissue. *p* values for overall survival (OS) and disease-free survival (DFS) were determined with the log rank test and given only for significant results. *n*, number of patients

To assess the effect of mRNA and DNA methylation levels of *TET* gene family members, we performed a retrospective analysis of 88 patients. The patients’ median survival was 38 (range 4–59) months. The transcript levels of TET1, TET2 and TET3 were divided into three groups: low, intermediate and high mRNA levels. The analysis of TET1 transcript levels in histopathologically unchanged tissues and TET2 in cancerous tissue did not reveal statistically significant results (Fig. 5a, c). However, compared to those with intermediate and high TET1 transcript levels, patients with low TET1 mRNA levels in cancerous tissues trended toward favorable DFS outcomes (Fig. 5a). Although these results did not reach statistical significance, low TET1 mRNA levels in cancerous tissue also correlated with increased patient OS, with a median survival of 41 months versus 36 and 33 months for intermediate and high TET1 mRNA levels, respectively (Fig. 5a). Moreover, follow-up data were available for eight out of 12 patients with DNA hypermethylation within CpG islands of the *TET1* gene in cancerous tissues. Log rank test analysis did not reveal statistically significant results for the patients with DNA methylation (Fig. 5b). Further, Kaplan–Meier analysis revealed that the CRC patients with high TET2 mRNA level in histopathologically unchanged tissues had better overall and DFS outcomes (OS median: 41 months; DFS median: 40 months) than those with low TET2 mRNA levels (OS median: 26.5 months; DFS median: 28 months) (Fig. 5c). In the present study, analysis of TET3 mRNA levels in histopathologically unchanged and cancerous colorectal tissues revealed no significant correlation with the patients’ OS and DFS (Fig. 5d).

We performed multivariable analysis for TET2 mRNA levels in histopathologically unchanged tissues and for TET1 mRNA level in cancerous tissues. In Cox proportional hazard regression analysis, the categorical variables introduced included age, gender, tumor localization and postoperative chemotherapy. In the current study, adjusted analysis did not reveal statistically significant results for TET1 mRNA levels in cancerous tissues (Table 4). However, multivariable analysis revealed that high TET2 mRNA levels in histopathologically unchanged tissues can be an independent prognostic factor for OS and DFS (Table 5).

**Table 4:** Multivariate analysis of TET1 transcript levels in cancerous tissue in patients with colorectal cancer

Variable	OS		DFS	
	HR (95 % CI)	*p*	HR (95 % CI)	*p*
TET1 mRNA				
Low	1		1	
Intermediate	2.48 (0.69–8.85)	0.15	5.4 (0.99–22.82)	0.064
High	0.90 (0.14–5.75)	0.91	2.03 (0.32–12.73)	0.45
Gender				
Female	1		1	
Male	0.95 (0.35–2.57)	0.92	1.72 (0.63–4.73)	0.29
Age				
Below 60	1		1	
Above 60	1.11 (0.32–4.06)	0.83	1.49 (0.41–5.36)	0.51
Therapy				
No	1		1	
Yes	1.14 (0.44–2.97)	0.78	1.63 (0.64–4.17)	0.31
Tumor localization				
Rectum	1		1	
Proximal	0.49 (0.16–1.54)	0.22	0.79 (0.27–2.31)	0.67
Distal	0.85 (0.28–2.56)	0.77	0.96 (0.34–2.75)	0.95

**Table 5:** Multivariate analysis of TET2 transcript levels in histopathologically unchanged tissue in patients with colorectal cancer

Variable	OS		DFS	
	HR (95 % CI)	*p*	HR (95 % CI)	*p*
TET2 mRNA				
Low	1		1	
Intermediate	0.34 (0.11–1.02)	0.055	0.51 (0.18–1.44)	0.20
High	0.091 (0.011–0.77)	0.028	0.21 (0.04–1.06)	0.059
Gender				
Female	1		1	
Male	0.78 (0.28–2.20)	0.64	1.84 (0.67–5.05)	0.23
Age				
Below 60	1		1	
Above 60	1.25 (0.35–4.40)	0.73	1.16 (0.36–3.72)	0.80
Therapy				
No	1		1	
Yes	1.19 (0.41–3.46)	0.75	1.46 (0.53–4.02)	0.46
Tumor localization				
Rectum	1	1		
Proximal	0.61 (0.19–1.96)	0.40	0.79 (0.26–2.36)	0.67
Distal	0.79 (0.25–2.43)	0.68	0.98 (0.34–2.79)	0.96

## Discussion

Identification of TET activity was a milestone in understanding the dynamic landscape of the epigenome. Although the precise role of TET in physiological processes and diseases necessitates further elucidation, recent data point to a role of TETs in maintaining 5-hmC at genes susceptible to hypermethylation in cancer (Putiri et al. 2014). Several reports have also found a correlation between reduced 5-hmC and poor patient prognosis in various cancers (Hsu et al. 2012; Lian et al. 2012; Liu et al. 2013; Orr et al. 2012). We have observed reduced TET1, TET2 and TET3 transcript levels in a cohort of 113 CRC patients. These data support previous observations of lower levels of TET mRNA in other types of solid cancers: reduced *TET1* expression had been found in the cancerous tissues of breast, prostate and hepatocellular carcinomas (Frycz et al. 2014; Liu et al. 2013; Muller et al. 2012); moreover, all three TET family members were downregulated at the mRNA level in breast cancer and melanoma (Hsu et al. 2012; Lian et al. 2012; Yang et al. 2013). Interestingly, we also observed reduced TET3 mRNA levels in the cancerous tissues from patients above 60 years of age, and in tumors localized in the proximal colon. The proximal colon, distal colon and rectum develop from different embryological origins, as reflected by their vascular supplies, glucose metabolism and antigen presentation (Distler and Holt 1997). Moreover, during carcinogenesis, epigenetic changes mostly occur at the proximal site, whereas genetic background of tumor is characteristic for distal site and rectum (Jass 2007). To date, several publications have reported the sub-site-specific expression of genes in CRC (Glebov et al. 2003; Papaxoinis et al. 2010; Rawluszko et al. 2011), supporting the differences we observed in the TET3 transcript levels in the proximal colon.

Furthermore, we investigated for the first time the prognostic importance of TET mRNA levels in CRC. Surprisingly, we observed that the patients with low TET1 mRNA levels in cancerous tissues trended toward favorable outcomes. However, this tendency was not observed in multivariate analysis. In earlier studies, decreased TET1 mRNA levels in breast cancer were associated with increased invasiveness and metastasis in vitro and in vivo (Hsu et al. 2012). Our results in CRC need to be validated in large, multicenter studies with extended follow-ups and be verified through molecular analyses. However, we detected that high TET2 mRNA levels in histopathologically unchanged tissues may be related to favorable outcomes. To the best of our knowledge, this is the first report presenting such a result in a solid tumor. Previously, the significance of *TET2* expression in carcinogenesis was studied particularly for lymphomas and leukemias. In *TET2* knockout studies, decreases in 5-hmC levels stimulated the self-renewal of hematopoietic stem cells and their altered development toward the monocyte lineage (Ko et al. 2011; Moran-Crusio et al. 2011). Moreover, loss-of-function mutations in *TET2,* which impair its catalytic activity, are often observed in patients with chronic myelomonocytic leukemia (CMML), myeloproliferative neoplasms (MPN), myelodysplastic syndrome (MDS), as well as B cell and T cell lymphomas (Asmar et al. 2013; Couronne et al. 2012; Delhommeau et al. 2009; Langemeijer et al. 2009).

Unlike *TET2*, loss-of-function mutations of *TET1* and *TET3* are uncommon. Moreover, according to the COSMIC database, mutations in *TET* family members in solid tumors are very rare (1–3 %). It should be noted that the *TET1*, *TET2* and *TET3* genes possess CpG islands in their promoter region. Thus, we investigated whether the reduction of the mRNAs in cancerous tissues might stem from aberrant DNA methylation. However, in our study group, no DNA methylation was observed in histopathologically unchanged and cancerous tissues at the *TET2* and *TET3* promoters. To date, *TET2* promoter hypermethylation had been observed for low-grade diffuse gliomas, a subset of Ph-negative MPN and pediatric acute lymphoblastic leukemia (ALL) (Chim et al. 2010; Musialik et al. 2014). The biological effects of *TET2* hypermethylation, as reflected by lower 5-hmC levels, had been observed in multiple sclerosis (Calabrese et al. 2014). On the contrary, DNA methylation in the *TET2* promoter was not detected in MPN, MDS or CMML (Abdel-Wahab et al. 2009; Jankowska et al. 2009). To the best of our knowledge, DNA methylation within the *TET3* promoter has not been reported, and our study presents its absence.

We found *TET1* DNA hypermethylation in cancerous tissues from a small subset of CRC patients (*n* = 12/113). The Mann–Whitney test revealed that compared to the group without DNA methylation, the group with DNA methylation showed lower *TET1* transcript levels in cancerous tissues. However, these findings should be interpreted with caution, due to potential bias arising from the sample size. Although *TET1* DNA methylation is a very interesting observation, the study should be extended to a larger group of patients to identify its biological significance and prognostic value. Consistent with our findings, other reports have also indicated the relevance of *TET1* promoter DNA methylation in cancer. *TET1* hypermethylation was observed in the ALL cell line SKW-3, and the cervical cancer cell line in HeLa (Ciccarone et al. 2014). Moreover, *TET1* promoter was methylated in high-mobility group AT-hook 2 (HMGA2; chromatin remodeling factor)-depleted breast cancer cells (Sun et al. 2013).

It is known that *TET* expression might be regulated posttranscriptionally or posttranslationally (Delatte et al. 2014). Hence, the main limitation of our study is the lack of correlation between TET protein levels and 5-hmC. Keeping in mind the small amount of samples, we decided to investigate *TET* DNA methylation and mRNA levels in CRC because it had not been previously explored.

In conclusion, our study demonstrates that TET1, TET2, and TET3 transcript levels in cancerous tissues are reduced and provide the first evidence that TET2 mRNA in histopathologically unchanged tissue from CRC patients may be an independent predictor of relapse and OS. Nevertheless, further large-scale, longitudinal studies are needed to confirm the clinical relevance of this observation. Moreover, DNA methylation within the *TET1* promoter region suggests it may play a role in regulating *TET1* gene expression. Nonetheless, in the context of CRC, this hypothesis still necessitates direct testing by additional in vitro and in vivo studies.

## Electronic supplementary material

Below is the link to the electronic supplementary material.

Supplementary material 1 (DOC 39 kb)
