# Supplementary material for: Clinical significance of DNA methylation mRNA levels of TET family members in colorectal cancer
Source: J Cancer Res Clin Oncol. 2015 Jan 4;141(8):1379–92. doi: 10.1007/s00432-014-1901-2 (PMC4469774; doi:10.1007/s00432-014-1901-2)
Supplement: Supplementary file 1 — Supplementary material 1 (DOC 39 kb) [file 432_2014_1901_MOESM1_ESM.doc]

| **Primers** | **Sequence (5’-3’)** | **UCSC position (GRCh37/hg19)** | **Product**  **size (bp)** |
| --- | --- | --- | --- |
| **Primers for RQ-PCR** | | | |
| TET1 | F: 5’ATACAATGGGCACCCTACCG3’ R: 5’GGGCTTGGGCTTCTACCAAA3’ | chr10:70 320 117-70 454 239 | 159 |
| TET2 | F: 5’GCTGACAAACTCTACTCGG3’ R: 5’CTTCTGGCAAACTTACATCC3’ | chr4:106 067 842-106 200 960 | 188 |
| TET3 | F: 5’CCCAAAGAGGAAGAAGTG3’ R: 5’GCAGTCAATCGCTATTTC3’ | chr2:74 273 405-74 335 302 | 129 |
| PBDG | F: GCCAAGGACCAGGACATC  R: TCAGGTACAGTTGCCCATC | chr11:118 468 348-118 468 864 | 160 |
| hMRPL19 | F: ACTTTATAATCCTCGGGTC  R: ACTTTCAGCTCATTAACAG | chr2:75 735 389- 75 735 705 | 171 |
| **Primers for bisulfite sequencing of CpG island** | | | |
| TET1 | F: TTTTTTATTGTGGATTTTTGGGA  R: CATTATTTATCTCCGACAACAAAAA | chr10: 70 320 271-70 320 716 | 446 |
| TET2 | F: TTTTTTTTTAGGGGTGGA  R: ACTTACATACGAACGAAACCC | chr4: 106 067 501-106 068 077 | 577 |
| TET3 | F: GGGTTTATTTGGATAGGGTATT  R:TACAATAACTCCCACCCAC | chr2: 74 211 418-74 211 829 | 412 |
| **Primers for HRM analysis of CpG island** | | | |
| TET1 | F: 5’TTTTTATTGTGGATTTTTGGGA3’ R: 5’CTCCAAACCTACACCAACCC3’ | chr10:70 320 271-70 320 457 | 187 |
| TET2 | F: 5’GGGGTTTTCGAGGGTTGTAT3’ R: 5’TCCCAAAAAATTAACCCCC3’ | chr4:106 067 537-106 067 735 | 199 |
| TET3 | F: 5’GGGTTTATTTGGATAGGGTATT3’ R: 5’AACCCCCCTATCCCAACT3’ | chr2:74 211 418-74 211 584 | 167 |

**Supplementary table 1. Primer sequences**F- forward R-reverse
